# Functional Analyses of the *Toxoplasma gondii* DNA Gyrase Holoenzyme: A Janus Topoisomerase with Supercoiling and Decatenation Abilities

**DOI:** 10.1038/srep14491

**Published:** 2015-09-28

**Authors:** Ting-Yu Lin, Soshichiro Nagano, Jonathan Gardiner Heddle

**Affiliations:** 1 Heddle Initiative Research Unit, RIKEN, 2-1 Hirosawa, Wako, Saitama, 351-0198, Japan

## Abstract

A number of important protozoan parasites including those responsible for toxoplasmosis and malaria belong to the phylum Apicomplexa and are characterised by their possession of a relict plastid, the apicoplast. Being required for survival, apicoplasts are potentially useful drug targets and their attractiveness is increased by the fact that they contain “bacterial” gyrase, a well-established antibacterial drug target. We have cloned and purified the gyrase proteins from the apicoplast of *Toxoplasma gondii* (the cause of toxoplasmosis), reconstituted the functional enzyme and succeeded in characterising it. We discovered that the enzyme is inhibited by known gyrase inhibitors and that, as well as the expected supercoiling activity, it is also able to decatenate DNA with high efficiency. This unusual dual functionality may be related to the apparent lack of topoisomerase IV in the apicoplast.

*T. gondii* is the single-cell parasite responsible for toxoplasmosis^1^. It is a major cause of death due to food-borne illness^2^ and a particular risk for pregnant women, where it can result in loss of pregnancy and illness in infants^2^, as well as for the immune compromised. The disease affects over one million people per year in the USA^3^ and drug treatments are relatively ineffective, with a risk of toxicity^1^. The parasite is a member of the phylum Apicomplexa most of which are characterised by their possession of apicoplasts. The apicoplast was discovered in the 1990s^4^ and is a four-membraned organelle that arose as the result of secondary endosymbiosis of a green or red alga, containing the plastid, itself the result of primary endosymbiosis of a cyanobacterium-related bacterium^5^. It contains a 35-kb circular plastid genome^6^ largely encoding rRNAs and ribosomal proteins amongst others^7^,^8^ and has its own transcription and translation machinery of bacterial origin^9^,^10^. It is involved in lipid, isoprenoid and haem synthesis^10^ but its full metabolic role is unclear. Other members of the phylum include *Plasmodium* spp. and *Eimeria* spp. With *Plasmodium*, the malaria parasite, being of particular interest due to its large toll on human health. The majority of apicoplastic proteins are encoded in the nuclear genome and are transported to the apicoplast due to the presence of signal and targeting peptides^10^. As the apicoplast is unique to Apicomplexa and is known to be vital to parasite replication and growth, it has been mooted as a potentially useful drug target^11^.

As a result of its bacterial origin and the possession of a circular genome, the apicoplast retains a “bacterial” DNA gyrase protein (“gyrase”)^12^. Gyrase belongs to the type II DNA topoisomerase (topo II) family, is required to regulate DNA topology and possesses the unique ability to negatively supercoil DNA. Gyrase consists of two proteins, GyrA and GyrB, which form the functional, A_2_B_2_ tetramer. The mechanism of gyrase action has been well studied and is known in some detail: GyrA contains the catalytic domain that cleaves one segment of double-stranded DNA through which a second strand is passed. The broken segment is then religated. A single round of negative supercoiling requires hydrolysis of two ATP molecules. The N-terminal domain of GyrB is responsible for ATP hydrolysis and dimerises when ATP binding occurs, capturing the second strand, while the C-terminal region of GyrB interacts with GyrA and DNA^13^. Negative supercoiling specificity is ensured by the C-terminal regions of GyrA^14^,^15^. Gyrase is an important antibacterial target, particularly for the fluoroquinolone class of drugs. These act by inhibiting the DNA religation step, ultimately leading to release of DNA double-strand breaks, genome fragmentation and cell death^16^.

Many bacteria also contain a second type II topoisomerase known as topoisomerase IV (topo IV^17^,^18^) which has a different and somewhat complementary function; most notably it is unable to carry out supercoiling and shows a preference for decatenation/relaxation of DNA. *Mycobacterium tuberculosis* is an unusual example of a species of bacteria lacking topo IV and it has been found that, in order to compensate for this absence, its gyrase (MtGyr) has substantial functional differences compared to gyrase from *E. coli* (EcGyr). Notably it has enhanced DNA relaxation and decatenation activities^19^.

The discovery of gyrase in Apicomplexa, combined with its absence from human cells, raised the possibility that gyrase-targeting drugs could be used as effective treatments for diseases caused by these pathogens^20^,^21^. Indeed, the fluoroquinolone, ciprofloxacin (CFX) was tested *in vitro* against *T. gondii* where it was found to be toxic and depleted the copy number of the plastid genome relative to the nuclear genome, suggesting that CFX preferentially targeted apicoplast gyrase^22^. However, the role and effectiveness of fluoroquinolones as anti-apicomplexan drugs is still unclear; while *in vitro* studies suggest they are effective against *T. gondii*^22^ and they also inhibit growth of *P. falciparum*^23^, the parasites are not immediately killed by this treatment. Instead, both *P. falciparum* and *T. gondii* exhibit a characteristic “delayed death” response where death occurs in the generations subsequent to exposure, after invasion of a new host, rather than in the individuals exposed to the drug^22^,^24^,^25^. While evidence points to gyrase as the target of these drugs, this has not been conclusively proved although the production of double-stand cleaved apicoplast DNA in the presence of CFX in *Plasmodium spp*^24^,^26^, a characteristic consequence of the formation of gyrase-DNA-fluoroquinolone complexes, is suggestive. Furthermore, (fluoro)quinolones appear to be ineffective in treating apicomplexan diseases *in vivo* with ciprofloxacin and norfloxacin having been tested^27^,^28^,^29^. This may be due to difficulty in accessing the interior of the apicoplast, which requires the traversal of at least seven membranes, or it may be due to structural/functional differences in the apicoplast gyrases themselves, making them resistant to the drugs. Certainly there appear to be major differences in both size and structure of apicomplexan gyrases compared to “standard” bacterial gyrases^30^.

These issues may only be fully resolved if apicomplexan gyrases can be cloned, purified and biochemically assessed for enzymatic activity and response to inhibitors. Unfortunately this has not yet proved possible. To date most efforts have concentrated on gyrase from *P. falciparum* and the full length GyrB protein from this organism can be cloned and purified^20^. Full length *P. falciparum* GyrA however, has proved resistant to attempts to produce and purify it, meaning that comprehensive biochemical testing of the functional enzyme has not been achieved.

In this work we demonstrate the first production, purification and characterisation of a complete apicomplexan gyrase and find it to have unusual properties compared to most other characterized gyrases. Notably, in addition to the expected supercoiling activity, which shows enhancement by potassium glutamate, it also has a high decatenation activity and reduced susceptibility to calcium-induced cleavage.

## Results and Discussion

### Production and purification of TgGyrA and TgGyrB

Putative genes predicted to encode the apicoplastic TgGyr proteins^31^ can be found in the ToxoDB^32^ and encode TgGyrA (ToxoDB number: TGGT1_221330) and TgGyrB (ToxoDB number: TGGT1_293260). As the genes for both proteins reside in the nuclear DNA, they must be imported into the apicoplast; this requires signal and transit peptides. Interestingly TgGyrB lacks a canonical signal/transit peptide but we were previously able to predict its likely sequence^30^. Both TgGyrA and TgGyrB protein constructs were produced based on these predictions, as mature proteins consisting of amino acids 259–1594 for TgGyrA and 346–1386 for TgGyrB.

Both proteins were successfully produced including a N-terminal 6×His-tag. The yield of TgGyrB was 16 mg from 1 L of culture whereas TgGyrA was only 0.02 mg from 4 L of culture. TgGyrA was predicted to be a 150 kDa protein while TgGyrB was predicted to be 115 kDa according to amino acid composition. The purified proteins were resolved using SDS-PAGE and the purity of TgGyrA and TgGyrB was estimated to be approximately 10% and 95%, respectively (Supplementary Fig. S1a). The identity of purified proteins was confirmed by MALDI-TOF (data not shown). The percentages of peptide sequences identified by MALDI-TOF analyses were 39% for TgGyrA and 59% for TgGyrB.

In order to increase the purity of TgGyrA, we attempted to purify it in the presence of TgGyrB which, as for the case of coexpression of *S. aureus* GyrA and GyrB^33^, is expected to assist in stabilizing the GyrA subunit and decreasing background contamination. TgGyrA- and TgGyrB-containing cells were grown separately and cell lysates were mixed before affinity and size exclusion purification. These co-eluted samples were resolved using SDS-PAGE and the TgGyr complex was estimated to be 37% of the total protein mixture by comparing the total intensities of the GyrA and GyrB bands on the SDS PAGE gel to all protein bands using ImageJ^34^ (Supplementary Fig. S1b). While this approach succeeded in significantly increasing the effective TgGyrA purity, the yield of the TgGyr complex was still very low, at 0.15 mg. In this report “TgGyr” refers to the co-eluted holoenzyme whereas “TgGyrA” and “TgGyrB” indicate the individually purified proteins.

### ATPase activity of TgGyrB

**Figure 1:**
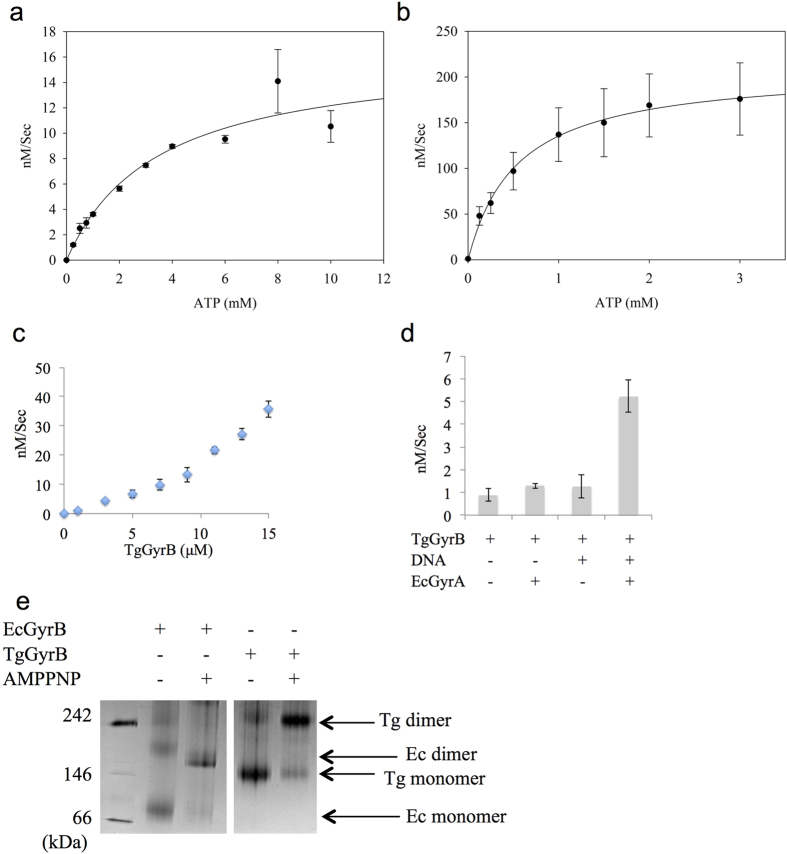
TgGyrB shows intrinsic ATPase activity and shares similar features with EcGyrB. (**a**) TgGyrB (4 μM) ATPase rate as a function of ATP concentration. (**b**) EcGyrB (1 μM) ATPase rate as a function of ATP concentration. The apparent Km of ATP was calculated as 0.54 ± 0.05 mM. (**c**) ATPase activity analysis of TgGyrB as a function of protein concentration. (**d**) ATPase activity of TgGyrB (2 μM) in response to the presence of the linear pBR322 DNA (2.8 nM) and EcGyrA (2 μM). (**e**) Native PAGE showing the oligomeric state of TgGyrB (2 μM) compared to EcGyrB (2 μM) in the presence and absence of AMPPNP. Samples were incubated at 25 °C. The native protein marker is shown in the leftmost lane. Experimental results were obtained from three independent data sets and standard deviations are shown.

An NADH-linked ATPase assay^35^ was performed to determine TgGyrB ATPase activity. The apparent Km was calculated as 3.04 ± 0.63 mM (Fig. 1a), similar to (approx. 3–5 fold higher) that reported previously for *E. coli* GyrB (see also Fig. 1b)^36^ and for the holoenzyme from *M. tuberculosis*^37^. However, as Fig. 1c shows, plotting the ATP hydrolysis rates of varying concentrations of TgGyrB resulted in a non-linear relationship, making Vmax and Km figures concentration-specific. A similar nonlinear dependence has been reported for EcGyrB (43 kDa N-terminal region)^35^ where it is thought to reflect the dimerisation of GyrB monomers upon ATP binding. This is also likely to apply to the full-length protein although less dramatically due to an increased propensity of the full-length protein to dimerise. The results for TgGyrB are in contrast to those reported for *Plasmodium falciparum* GyrB (PfGyrB), which shows a linear dependence of ATP hydrolysis rate on protein concentration, something that was attributed to the protein existing predominantly in the dimer form^20^. The reasons for this difference and implications for enzyme mechanism are currently unclear.

The results also show that TgGyrB has a low intrinsic ATPase activity, requiring considerably greater protein concentrations to achieve ATP turnover rates comparable to EcGyrB. A low ATP turnover rate has also been observed in MtGyrB^37^ and may be a reflection of slower growing organisms^38^.

The ATPase rate of TgGyrB demonstrated clear DNA stimulation (in the presence of EcGyrA) with ATP hydrolysis activity enhanced five-fold compared to that of TgGyrB alone (Fig. 1d). Enhancement of ATPase activity when in complex with EcGyrA and DNA^39^,^40^ is also a characteristic of EcGyrB and further similarities between TgGyrB and EcGyrB were shown by the fact that addition of DNA alone to TgGyrB had no significant effect on ATPase activity. Similarly, no effect on ATPase activity was observed for the EcGyrA-TgGyrB complex in the absence of DNA (Fig. 1d).

An indication of the functional similarity between gyrases including similar/compatible interacting regions can be obtained by protein swapping experiments in which the GyrA protein from one species is complexed with the GyrB protein from another and vice versa^20^. When these experiments were carried out using *Tg* and *Ec* proteins, EcGyrA-TgGyrB showed limited supercoiling suggesting that TgGyrB functions as a true GyrB (Supplementary Fig. S2). This result also allowed us to overcome limitations associated with the fact that only small amounts of TgGyrA could be purified (i.e. TgGyrB ATPase experiments could be carried out with EcGyrA replacing TgGyrA).

### TgGyrB dimerises in the presence of nucleotide

As the ATPase results were indicative of TgGyrB existing as a monomer rather than dimer in solution, we investigated its oligomeric state using non-denaturing PAGE (Fig. 1e). TgGyrB showed a slightly (+~ 23%) larger than theoretical molecular weight (~141 kDa compared to ~115 kDa) (Fig. 1e). The effect of nucleotide binding on oligomeric state was monitored by addition of AMPPNP followed by a 15 min incubation. In the presence of the nucleotide analogue the TgGyrB band moved position to an apparent molecular weight of ~275 kDa. Western blotting was then performed and confirmed that the observed band was His-tagged TgGyrB (Supplementary Fig. S3). This dimerisation pattern (monomer in the absence of nucleotide, dimer in the presence) is the same as for EcGyrB.

Overall these results, in combination with ATPase results, suggest that TgGyrB is similar to EcGyrB in terms of monomer-dimer equilibrium. These findings are supported by the observation that in EcGyrB, Ile10 is required for the dimerisation process with the involvement of Tyr5^41^. Both residues are retained in TgGyrB as well as PfGyrB (Supplementary Fig. S4) although other mutations of the equivalent of Ile10 in PfGyrB do not affect dimerisation (in the absence of ATP) suggesting that other residues play important roles^41^.

### DNA supercoiling and relaxation activities of the TgGyr holoenzyme

**Figure 2:**
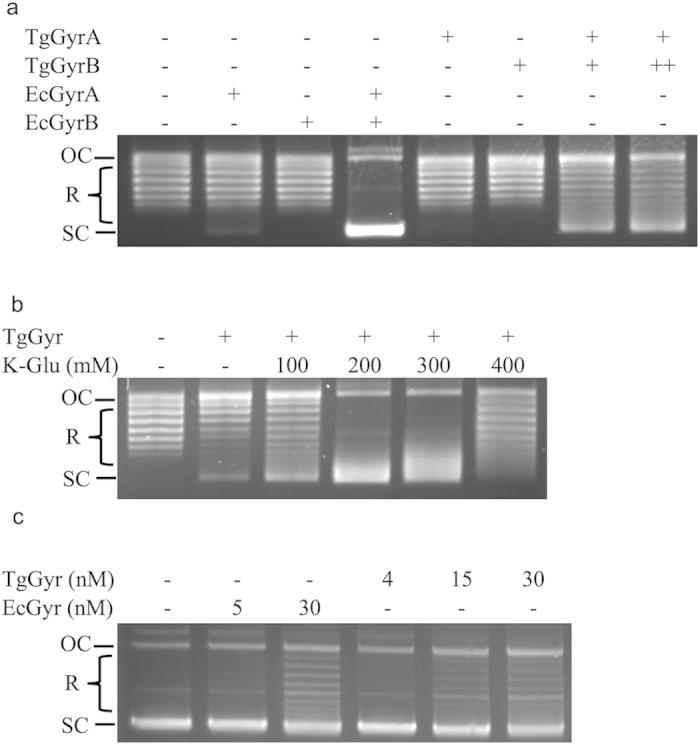
DNA supercoiling and relaxation activities of TgGyr. (**a**) DNA supercoiling assays were performed with relaxed pBR322 DNA (0.5 μg) and proteins as indicated at 37 °C for 30 min. Reconstituted TgGyr [TgGyrA: 6 nM (+) and TgGyrB: 18 nM (+) or 36 nM (++)] and EcGyr (33 nM: labeled as +) holoenzymes were used. The reactions were carried out in the presence of 100 mM K-Glu and 1 mM ATP. (**b**) DNA supercoiling activity assays were performed as in (**a**) but using different concentrations of K-Glu and 6 nM of TgGyrA and 18 nM of TgGyrB. (**c**) DNA relaxation activity of TgGyr compared to EcGyr. Assays were performed with proteins and supercoiled pBR322 DNA (0.4 μg) in the absence of ATP at 37 °C for 60 min. All DNA samples were resolved using a 1% agarose gel. The TgGyr used was the copurified holoenzyme. OC: Open circular DNA; R: Relaxed topoisomers; SC: Supercoiled DNA.

DNA supercoiling assays were performed to test TgGyr holoenzyme activity. TgGyrA (6 nM) and TgGyrB (18 nM and 36 nM) were mixed together and the results showed that the formed complex had the ability to supercoil DNA although with a rather low activity (Fig. 2a). Incubation of TgGyrA or TgGyrB alone with reaction mixture resulted in no observed supercoiling activity, showing that the activity seen was not due to contaminating gyrase from *E. coli*. However, the TgGyr exhibited a lower supercoiling activity comparing to EcGyr. MtGyr similarly shows low rates of supercoiling that are associated with truncation of the GyrA “tail”^42^. In contrast TgGyrA retains a more complete sequence in terms of number of amino acids compared to MtGyrA but this sequence is basic compared to the acidic tail found in other GyrAs (Supplementary Fig. S5). This change in charge characteristics in TgGyrA may well also explain its reduced supercoiling and relaxation activities.

Potassium chloride and potassium glutamate (K-Glu) are known to be required for DNA decatenation by topo IV^43^,^44^ but inhibit DNA relaxation by the same^33^,^45^. It is also known to differentially affect the supercoiling activity of gyrases from different species^33^,^46^ and is thought to play a role in DNA wrapping^47^. Stimulation of DNA supercoiling activity by K-Glu is observed in *S. aureus*^33^, *M. tuberculosis*^44^ and *S. pneumoniae*^48^ DNA gyrases but only minor effects are seen for EcGyr^46^. For example, 400–700 mM K-Glu is required for full supercoiling activity of *S. aureus* gyrase whereas 100–200 mM is optimal for EcGyr supercoiling activity^46^. There are several possible reasons to explain the effect of K-Glu on gyrase activities. In the case of *S. aureus* gyrase, the specific enhancement of supercoiling seen in the presence of K-Glu is likely due to a requirement for a high concentration of the salt to mediate binding to the GyrA C-terminal DNA wrapping domains^47^. In *S. aureus* cells, the concentration of glutamate is indeed higher than in *E. coli* (by approximately one order of magnitude)^49^,^50^,^51^.

To investigate the effect of K-Glu on the supercoiling reaction of TgGyr we performed the reactions with different concentrations of K-Glu (0–400 mM). Supercoiling efficiency of TgGyr was enhanced by K-Glu at 100 and 200 mM (Fig. 2b). At 400 mM K-Glu, supercoiling activity was abolished.

We also tested the relaxation activity of TgGyr as high concentrations of EcGyr in the absence of ATP are known to be able to relax supercoiled DNA substrates. We found that TgGyr is able to relax DNA although the activity appears even lower than for EcGyr (Fig. 2c).

**Figure 3:**
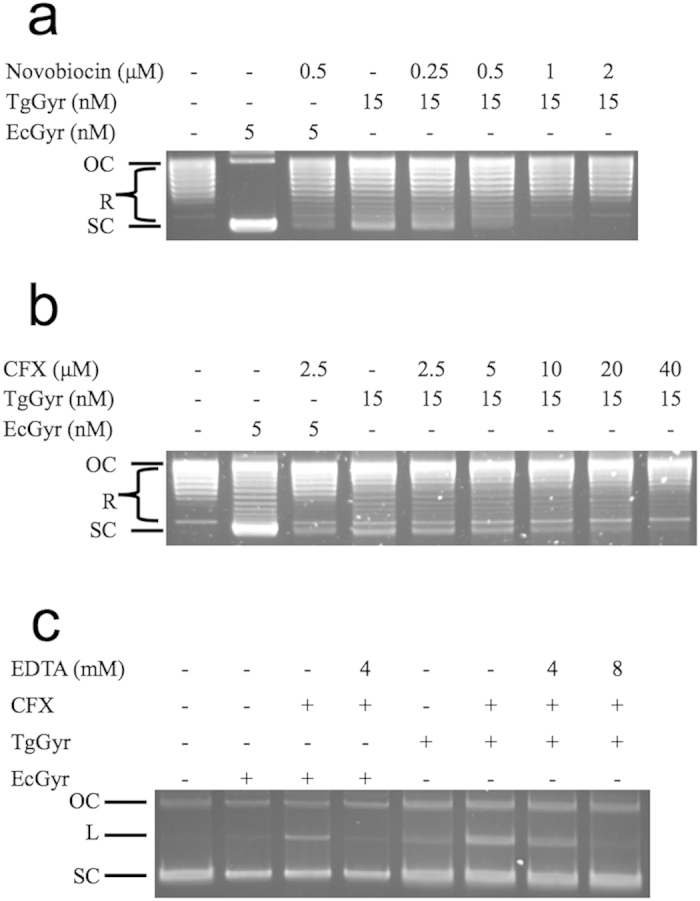
The inhibitory effects of novobiocin and ciprofloxacin (CFX) on TgGyr activity. (**a**) Effect of novobiocin on DNA supercoiling. Reactions were performed with relaxed pBR322 DNA (0.5 μg) and proteins and inhibitors as indicated at 37 °C for 30 min. EcGyr was 5 nM whereas the TgGyr was 15 nM. (**b**) Effect of CFX on DNA supercoiling. The reactions were carried out as in (**a**). (**c**) Gyrase-mediated DNA cleavage assays were performed with supercoiled pBR322 DNA (0.3 μg) and the presence/absence of CFX (5 μM) and EDTA as indicated at 37 °C for 60 min. ATP was omitted in these reactions. Samples with EDTA were incubated for an additional 30 min after EDTA addition. Reactions were stopped by adding SDS and proteins were removed by treatment with proteinase K at 56 °C for 60 min. The samples were resolved on a 1% agarose gel. OC: Open circular DNA; L: Linear DNA; SC: Supercoiled DNA.

Next, we tested if the supercoiling reaction of the TgGyr complex was sensitive to common gyrase inhibitors. We found that novobiocin inhibited supercoiling activity (Table 1, Fig. 3a) with complete inhibition observed at 1 to 2 μM and an IC50 of approx. 0.3 μM. CFX also exhibited an inhibitory effect toward TgGyr (Table 1, Fig. 3b) but with an apparent IC50 of 7.7 μM that was approx. 10-fold higher than for EcGyr (IC50 = 0.8 μM).

Both novobiocin and CFX inhibited the supercoiling reaction of TgGyr at similar concentrations to those seen for MtGyr^52^ (Table 1). In the case of CFX, this is higher than the concentrations required to inhibit supercoiling by other bacterial gyrases such as EcGyr^53^. For MtGyr this was attributed to the fact that the equivalent position to EcGyrA Ser83, an important residue for quinolone binding in the quinolone-resistance determining region (QRDR) of MtGyr is an alanine (Ala90)^54^. Alignment of the TgGyrA amino acid sequence with MtGyrA and EcGyrA (Supplementary Fig. S6, S7a) shows that TgGyrA also lacks Ser at the equivalent of position 83, having instead a Gln (residue 348, full-length numbering).

These results suggest that, in TgGyr, the ATP-binding site and the active site are highly similar to that of bacterial enzymes (as implied by sequence homology^30^) and furthermore that the additional sequence/domains in the apicomplexan enzyme do not act to shield the binding sites from the effect of drugs, resulting in apparent Kds in a similar range to those for bacterial enzymes; The exception being classical fluoroquinolones due to the presence of residue Gln348.

While the effectiveness of novobiocin in inhibiting TgGyr indicates a similar ATP binding-hydrolysis site to known gyrases and offers a potential drug target, for the QRDR and fluoroquinolone binding the story is more complex: If the *in vivo* and *in vitro* pattern of MtGyr is followed^19^, we would expect the general reduced susceptibility to fluoroquinolones observed in the *in vitro* results presented in this work. Tests of various quinolones (including ciprofloxacin) against *T. gondii* have suggested poor activity (IC50s above 10 mg/ml) for the majority with trovafloxacin, grepafloxacin, gatifloxacin, and moxifloxacin being potent growth inhibitors amongst those tested in culture^55^.

Resistance to quinolones observed *in vivo* may also be due to additional factors unique to apicomplexan gyrases such as access of the drug molecules to the enzymes. Given the apparent effectiveness of some fluoroquinolones against *T. gondii* and the proven worth of gyrase as a drug target, it may be useful to consider development of systems for delivering fluoroquinolones to the apicoplast.

### The CFX-induced DNA cleavage activity of the TgGyr holoenzyme

The higher concentration of CFX required to inhibit DNA supercoiling by TgGyr in conjunction with the presence of a quinolone resistance mutation in the QRDR strongly suggests the presence of a quinolone binding pocket in TgGyr similar to that found in other, characterised gyrases. CFX is known to function by stabilization of the “cleavage complex” consisting of gyrase and double strand cleaved DNA^56^. Stabilisation of the cleavage complex was investigated for TgGyr and CFX and was shown to induce DNA cleavage as evidenced by production of linear DNA (Fig. 3c). Addition of EDTA is known to reverse gyrase cleavage complex formation allowing cleaved DNA to religate. Addition of EDTA to the reaction was performed to test if the cleavage was reversible in the case of TgGyr. Results showed that EDTA led to a decrease in the amount of cleaved (linear) DNA produced (Fig. 3c), again suggesting commonality in the structure of the fluoroquinolone binding pocket between TgGyrA and other, characterised gyrases.

### The DNA decatenation activity of TgGyr

**Figure 4:**
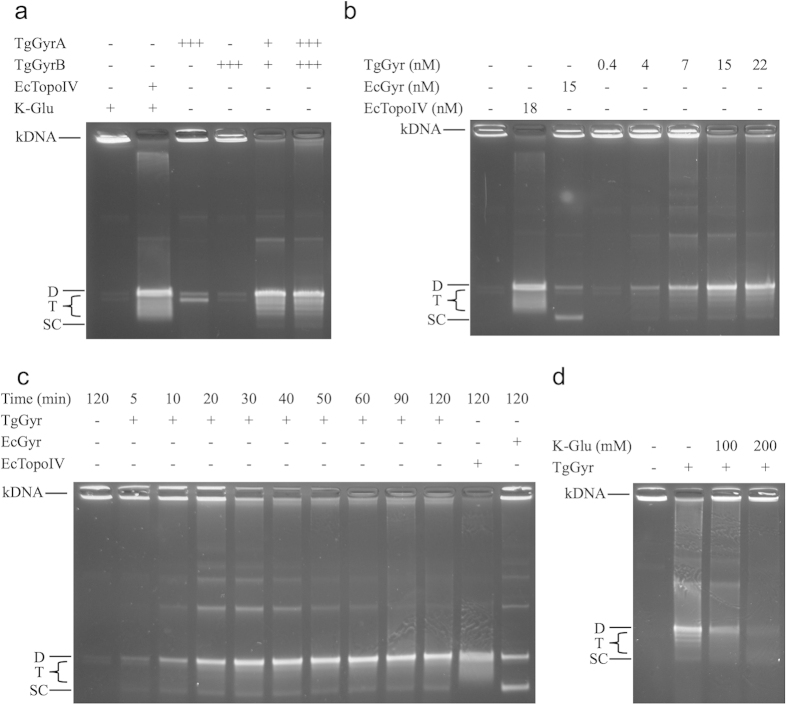
Decatenation activity of TgGyr. (**a**) DNA decatenation activity assays were performed with proteins as indicated and kDNA (0.2 μg) at 37 °C for 30 min and DNA was resolved on a 1% agarose gel. Reconstituted TgGyr (10 and 30 nM: labeled as + and +++ respectively) and topoIV (18 nM: labeled as +) holoenzymes and the equivalent concentrations of TgGyrA and TgGyrB were analyzed. Reactions were carried out in the presence of 1 mM ATP. (**b**) DNA decatenation activity assays were performed as in (**a**) but incubated with copurified TgGyr. (**c**) Time course of TgGyr catalyzed DNA decatenation. (**d**) The effect of K-Glu on TgGyr decatenation. D: Decatenated substrate; T: Topoisomers; SC: Supercoiled DNA.

DNA decatenation is not a reaction favoured by EcGyr and EcTopo IV is required for this process during replication^57^. A decatenation assay was performed to investigate if TgGyr has decatenase activity (Fig. 4a). When TgGyrA and TgGyrB were incubated alone with reaction mixture, neither of them showed significant decatenation activity. In contrast, the reconstituted TgGyr complex completely decatenated kDNA into single circular molecules at concentrations as low as 10 nM. The co-eluted TgGyr complex also showed decatenation activity (Fig. 4b). It is interesting to note that the dual decatenation and supercoiling activities of TgGyr are demonstrated by the fact that the decatenated DNA in the decatenation reaction is used as a substrate for supercoiling as shown in Fig 4b. A similar result has been observed for MtGyr^44^,^58^. However, as the amino acid sequence alignment (Supplementary Fig. S6) shows, TgGyr is much larger than MtGyr suggesting that either it achieves the same result via a different mechanism or via the same mechanism in which case the extra residues in TgGyr have an unrelated, unknown function. In topoisomerase IV, the preference for decatenation is achieved via the C-terminal domains of ParC which have a different structure and orientation compared to their counterparts in gyrase^59^. This may also be the case for TgGyr where GyrA appears substantially different from other bacterial counterparts^30^.

Time courses of TgGyr decatenation and subsequent supercoiling activities (Fig. 4c) showed that a relaxed circular, decatenated DNA band appeared after 5 min incubation and the kDNA substrate was completely decatenated at 40 min. However the supercoiled band remained as only a minority of the product, consistent with the relatively low supercoiling activity previously observed (see Fig. 2).

We also tested whether K-Glu could enhance decatenation activity as it does supercoiling activity (Fig. 4d). Surprisingly, K-Glu at 100 mM inhibited TgGyr decatenation activity, abolishing it completely at 200 mM.

### The effect of calcium ions on TgGyr activities

**Figure 5:**
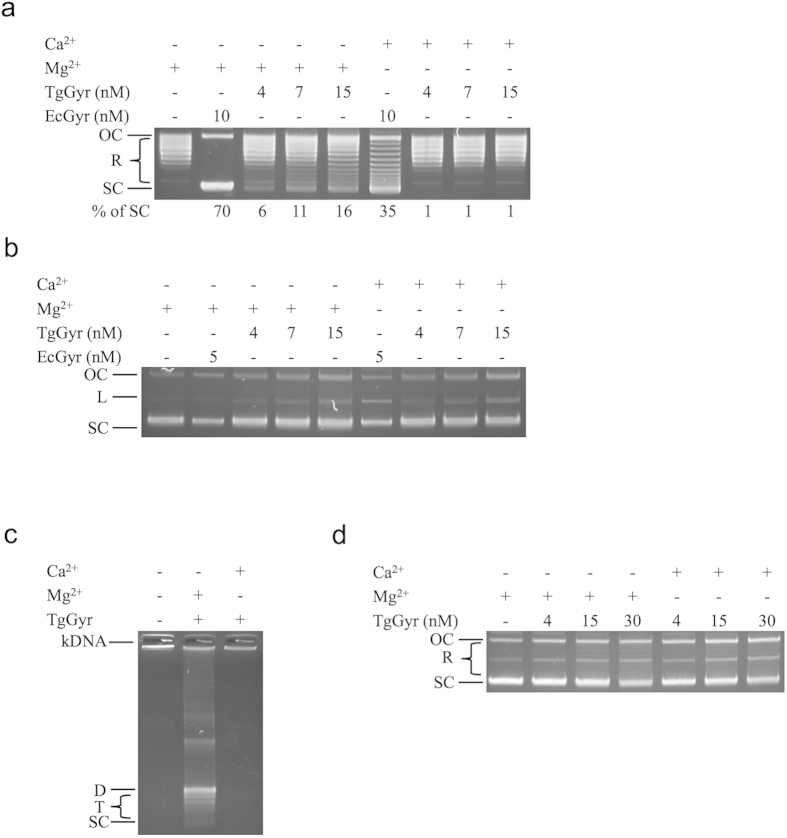
The effects of Ca^2+^ on TgGyr activities. (**a**) DNA supercoiling reactions were performed with proteins and relaxed pBR322 DNA (0.5 μg) and the presence of 6 mM of Mg(CH_3_COO)_2_ or CaCl_2_ at 37 °C for 30 min. The % of supercoiled DNA was quantified. (**b**) Gyrase-mediated DNA cleavage assays were performed with proteins and supercoiled pBR322 DNA (0.3 μg) and the presence of 6 mM of Mg(CH_3_COO)_2_ or CaCl_2_ at 37 °C for 60 min. ATP was omitted in these reactions. (**c**) DNA decatenation activity assays were performed with TgGyr (15 nM) and kDNA (0.2 μg) at 37 °C for 30 min. The effect of Ca^2+^ on TgGyr decatenation activity was examined. (**d**) DNA relaxation activity assays were performed with TgGyr and supercoiled pBR322 DNA (0.4 μg) in a reaction containing magnesium. An additional 1 mM CaCl_2_ was added as labeled. D: Decatenated substrate; L: Linear DNA; T: Topoisomers; SC: Supercoiled DNA.

In a recent study by Karkare *et al*., evidence for the existence of a calcium binding site in MtGyrA was presented as well as the fact that, in contrast to EcGyr, calcium ions cannot substitute for magnesium ions in supporting MtGyr supercoiling activity but favour relaxation and decatenation^60^. Given some apparent similarities between MtGyr and TgGyr including the fact that both appear to exist in environments without topo IV, we examined the effect of calcium on TgGyr activities by performing supercoiling assays in the absence of either magnesium or calcium (Fig. 5a) and found that replacing magnesium ions with calcium ions abolished supercoiling activity.

Calcium ions are also known to induce DNA cleavage by EcGyr but not by MtGyr^60^. We performed DNA cleavage assays in the presence of either magnesium or calcium (Fig. 5b). For TgGyr, the presence of Ca^2+^ did not result in the production of any additional linear DNA. Furthermore, calcium did not support TgGyr in decatenation reactions (Fig. 5c) and did not enhance TgGyr-mediated DNA relaxation (Fig. 5d).

The effects of Ca^2+^ on MtGyr have been attributed to a potential special regulatory role in controlling the supercoiling/decatenation balance. The probable binding site for Ca^2+^ in MtGyrA^60^ is not found in TgGyrA (Supplementary Fig. S7b) and the role of the ion appears different as it does not support decatenation activity, suggesting that the supercoiling/relaxation/decatenation balance is achieved by a different mechanism, again hinting at a possible role for the additional amino acids in TgGyrA, particularly at the C-terminus^30^. The possibility that Ca^2+^ has a similar effect on TgGyr as on MtGyr under buffer conditions different from those tested in this work cannot, of course, be ruled out.

### TgGyrA contains a potential GyrA-box

Given that DNA supercoiling activity was enhanced by the presence of K-Glu, we analysed the amino acid sequence of TgGyrA and found evidence that it may contain a second GyrA-box, as has been shown for MtGyrA^58^. In MtGyrA this is found in the β pinwheel, has the sequence QGRGGKG conforming to the consensus sequence (QXRGGK/RG) and is thought to have a role in enhancing decatenation activity^58^. It may function by promoting a unique DNA binding conformation that enhances intermolecular DNA binding^58^.

We carried out sequence alignments between EcGyrA, MtGyrA and TgGyrA (Supplementary Fig. S6 and S7c) and showed that TgGyrA has a degenerate GyrA-Box-1 sequence (QRRRGVG where underlined residues deviate from the consensus). The first deviation, R in place of G has been addressed in previous experimental work in MtGyr where replacement (with an alanine, G746A) had little effect on supercoiling or cleavage activity and resulted in decreased decatenation activity^58^. Taken with the effects observed for K-Glu, this could point to a mechanism of supercoiling control similar to that in MtGyr where the enhancement of supercoiling activity by K-Glu was attributed, at least in part, to an effect on DNA binding to the GyrA-box of the CTD^58^. However, a significant difference in the effect of K-Glu on TgGyr compared to MtGyr was observed in decatenation; it appeared to inhibit the decatenation activity of TgGyr rather than having the stimulatory effect seen for MtGyr^44^.

## Conclusion

Apicomplexan gyrases are unique enzymes, which differ significantly from the more familiar bacterial gyrases and are potentially useful therapeutic targets. To date however, they have been poorly characterised. In this work we have successfully produced and carried out the first characterisation of an apicomplexan gyrase holoenzyme.

Taken together, these results paint a picture of TgGyr as an unusual gyrase: its GyrB protein appears very similar to the GyrBs of EcGyr and other bacterial gyrases in terms of biochemical characteristics and is also closer to them in size compared to TgGyrA^30^. In contrast, TgGyrA is known to be very different at the sequence level due to the presence of a large amount of extra sequence and possible extra domains^30^. This difference may account for the deviation in characteristics of TgGyr from EcGyr. The high decatenation activity of TgGyr is a feature shared with MtGyr, consistent with the fact that both enzymes are likely the sole type II topoisomerases in their respective compartments. However given the dissimilar responses to Ca^2+^ it seems probable that the balance in activity between supercoiling and decatenation is achieved via a different mechanism in TgGyr. It remains to be seen if these findings apply to other apicomplexan gyrases and further biochemical and structural studies will be required to determine of these unique features can be exploited for the production of anti-apicomplexan drugs.

## Methods

Supercoiled, relaxed, linear pBR322 plasmid DNAs as well as kDNA were purchased from Inspiralis (Norwich, UK). AMPPNP, SYBR Gold and NativeMark™ unstained protein standard were purchased from Thermo Fisher Scientific (MA, USA). Proteinase K was purchased from Nacalai Tesque (Kyoto, Japan).

### Protein expression and purification

The TgGyrA (NCBI sequence identifier gi|523571679) and TgGyrB (NCBI sequence identifier gi|523571454) full length sequences were synthesized and cloned into pET28a (Genscript Inc., USA), producing plasmids pET28a(+)_TgGyrA and pET28a(+)_TgGyrB respectively. Residues 1–258 form the signal/transit peptide of TgGyrA^30^. As the mature protein is not expected to include this sequence it was omitted. The sequence without signal peptide was PCR amplified and cloned into pET28a(+) to produce pET28a(+)_TgGyrA_S_del. In our previous research, sequence alignments showed an extra N-terminal region (1–345) in the TgGyrB gene sequence. While the region was not predicted as a signal peptide this appears to be its likely function and we made a deletion of this region with the modified gene being cloned into pET28a(+)_TgGyrB_S_del.

Plasmids were transformed into BL-21 (DE3) competent cells. The cells were inoculated into 10 ml LB broth and incubated overnight. One milliliter was then transferred to 1 L LB broth for further incubation until the OD600 reached 0.5–0.7 (approximately 3.5 hrs). IPTG (0.25 mM) was then added to induce protein production. Protein induction was carried out overnight at 16 °C with agitation. The cells were collected by centrifugation at 6,000 rpm for 15 min at 4 °C (rotor JLA 9.1, Beckman Coulter). The cell pellet was then suspended in 50 mM Tris·HCl buffer (pH 7.5) containing 20 mM imidazole, 150 mM NaCl, 0.1% triton X-100, lysozyme (1 mg/ml), and protease inhibitor (Roche). Cell suspension was sonicated on ice and the cell debris was removed by centrifugation at 18,000 rpm for 2 h at 4 °C (rotor JA-20, Beckman Coulter). The supernatant was then loaded onto a HisTrap column (FF 5 ml, GE Healthcare) and an increasing gradient of elution buffer [50 mM Tris·HCl buffer (pH 8.0) containing 500 mM imidazole and 150 mM NaCl] was applied to elute His-TgGyrB. The eluate was concentrated using a spin column (cutoff: 10 kDa, Millipore) at 5,000 g at 4 °C and applied to a Superdex-200 column (10 × 30 HR; GE Healthcare) for size exclusion chromatography (SEC). The SEC buffer contained 50 mM Tris·HCl (pH 8.0), 150 mM NaCl, 1 mM dithiothreitol (DTT) and 1 mM EDTA with addition of 10% glycerol. The eluted protein was collected using an AKTA FPLC system (GE Healthcare Life Sciences), concentrated and then stored at −80 °C. The purification of EcGyrA and EcGyrB was carried out following the published method^61^.

Genes encoding ParC and ParE from *E. coli* were synthesized and cloned into pET28a(+) at BamHI and NdeI restriction sites (Genscript). Both proteins were produced and purified as described above. In brief, induction was carried out at 37 °C for 3 hrs followed by purification using a HisTrap column (FF 5 ml, GE Healthcare) and SEC. Both EcParC and EcParE were stored at −80 °C in buffer [40 mM HEPES·KOH buffer (pH 7.6), 100 mM K-Glu, 1 mM DTT and 1 mM EDTA with addition of 40% glycerol]^62^.

For purification of the TgGyr holocomplex, a modified method was used as follows: TgGyrA-containing cell lysate (from 6 L culture) was mixed with TgGyrB-containing cell lysate (from 0.5 L culture). The purification procedure as described above was then followed. In the last SEC elution step, fractions shown to contain both TgGyrA and TgGyrB by SDS PAGE analysis were collected, concentrated and then stored at −80 °C.

### ATPase assays

ATPase assays were performed using an ATPase enzyme-coupled method^35^ carried out in a 100 μl reaction volume in which the TgGyrB or EcGyrB enzymes were incubated in the reaction buffer [50 mM Tris·HCl (pH 7.5), 1 mM EDTA, 10% (w/v) glycerol, 25 mM KCl, 4 mM DTT, 5 mM MgCl_2_, 800 μM phosphoenolpyruvate, 400 μM NADH, 1% (v/v) PK/LDH (pyruvate kinase-lactate dehydrogenase mixture in 50% (w/v) glycerol, 100 mM KCl, 10 mM HEPES, pH 7.0)] and 2 mM ATP. The incubation was carried out at 25 °C for 10 min in a 96-well plate. The reaction was then started by the addition of 6.7 μl of 30 mM ATP and the change in OD340 nm was recorded over 10 min using a microplate reader [SpectraMax M2 (Molecular Devices, Sunnyvale, CA, USA)]. The ATP hydrolysis rate (nM/sec) was calculated from the first 5 min of readings using the equation: ∆340 nm/∆sec/6.22 mM^−1^ cm^−1^ × 1000000 where the 6.22 mM^−1^ cm^−1^ is the molar extinction coefficient of NADH at OD340 nm. Results were obtained from three independent data sets.

### Native gel analysis of TgGyrB dimerization

The proteins (2 μM) were incubated with or without 4 mM of AMPPNP in 10 μl reactions at 25 °C for 15 min. The reaction buffer contained 40 mM Tris·HCl (pH 7.5), 25 mM KCl, 6 mM Mg(CH_3_COO)_2_, 2 mM spermidine, 4 mM DTT, 0.36 mg/ml bovine serum albumin (BSA), 100 mM K-Glu and 0.1 mg/ml yeast tRNA. For analysis using the BN-PAGE technique^63^, the samples were loaded onto a 4%–16% NativePAGE^TM^ gel (Invitrogen) and resolved by electrophoresis at 8 °C according to the manufacturer’s instructions. The gel was stained with Coomassie blue and the protein bands were visualised. Gel images were obtained using a LAS3000 imager (Fujifilm, Tokyo, Japan) and analyzed by ImageJ^34^.

### DNA supercoiling assays

DNA supercoiling assays were performed in 30 μl at 37 °C for 30 min. Relaxed pBR322 DNA (0.5 μg) was mixed with enzymes in supercoiling buffer containing 40 mM Tris·HCl (pH 7.5), 25 mM KCl, 6 mM Mg(CH_3_COO)_2_, 2 mM spermidine, 4 mM DTT, 0.36 mg/ml bovine serum albumin (BSA), 100 mM K-Glu, 0.1 mg/ml yeast tRNA and 1 mM ATP. The concentration of enzymes is as specified in figure legends or labels. The reaction was stopped by adding an equal volume of STEB [40% sucrose, 100 mM Tris·HCl (pH 8.0), 10 mM EDTA and 0.5 μg/ml bromophenol blue]. The DNA substrates were then extracted by adding 30 μl of chloroform:isoamylalcohol (24:1), with vortexing followed by centrifugation at 14,000 rpm for 1 min. The aqueous phase containing DNA samples was applied to a 1% agarose gel. For experiments where samples contained K-Glu, samples were added to the wells of the gel 30 minutes prior to application of a voltage. Samples were then resolved by electrophoresis in 1X TAE buffer at 100 V for 60 min. The gel was stained by SYBR Gold and visualised using UV light. The image was then processed and analyzed using ImageJ^34^.

For the investigation of the ability of CaCl_2_ to substitute for Mg(CH_3_COO)_2_ in the TgGyr DNA supercoiling reaction, 6 mM of Mg(CH_3_COO)_2_ was replaced by 6 mM of CaCl_2_. Reactions were incubated at 37 °C for 30 min. DNA extraction was performed as described above. DNA samples were resolved on a 1% agarose gel by electrophoresis in 1X TAE buffer.

### DNA relaxation assays

DNA relaxation assays were performed as for DNA supercoiling assays but omitting ATP and spermidine and using supercoiled pBR322 DNA in place of relaxed DNA. The reaction contained 0.4 μg of supercoiled DNA and was carried out at 37 °C for 60 min. The extraction of DNA was performed as described above. DNA samples were resolved on a 1% agarose gel by electrophoresis in 1X TAE buffer.

### Decatenation Assays

A typical DNA decatenation assay was performed in 30 μl at 37 °C for 30 min. kDNA (0.2 μg) was used as the substrate and mixed with enzymes in supercoiling buffer but omitting K-Glu. Extraction of DNA was performed as described above. DNA samples were resolved on a 1% agarose gel by electrophoresis in 1X TAE buffer. For time courses, samples were removed for analysis at time points throughout the reaction.

### CFX-induced and calcium-induced DNA cleavage assays

DNA cleavage assays were performed as for DNA supercoiling assays but omitting ATP and using supercoiled pBR322 DNA (0.3 μg) instead of relaxed DNA. In calcium-induced cleavage experiments, 6 mM of Mg(CH_3_COO)_2_ was substituted with 6 mM of CaCl_2_. The reactions were carried out at 37 °C for 60 min. For reversibility tests, EDTA was added to the reaction which was incubated for an additional 30 min at 37 °C. Three microliters of 2% SDS and 1 μl of proteinase K (>700 U) were added to samples which were incubated at 56 °C for 60 min to denature proteins and remove them from DNA. Samples were extracted, resolved and analyzed as described above.

## Additional Information

**How to cite this article**: Lin, T.-Y. *et al.* Functional Analyses of the *Toxoplasma gondii* DNA Gyrase Holoenzyme: A Janus Topoisomerase with Supercoiling and Decatenation Abilities. *Sci. Rep.* **5**, 14491; doi: 10.1038/srep14491 (2015).

## Supplementary Material

Supplementary Information

## Figures and Tables

**Table 1:** IC50s of various drugs on gyrase supercoiling for gyrases from *E. coli*, *M. tuberculosis* and *T. gondii*.

**IC50 (μM) for gyrase supercoiling**			
	*Ec*	*Mt*	*Tg*
Novobiocin	0.3	1^52^	0.3
CFX	0.8	10^52^	7.7
