# Supplementary material for: Functional Analyses of the Toxoplasma gondii DNA Gyrase Holoenzyme: A Janus Topoisomerase with Supercoiling and Decatenation Abilities
Source: Sci Rep. 2015 Sep 28;5:14491. doi: 10.1038/srep14491 (PMC4585971; doi:10.1038/srep14491)

Functional Analyses of the *Toxoplasma gondii* DNA Gyrase Holoenzyme: A Janus Topoisomerase with Supercoiling and Decatenation Abilities

Ting-Yu Lin[1], Soshichiro Nagano[1#], and Jonathan Gardiner Heddle[1,*]

[1]Heddle Initiative Research Unit, RIKEN, 2-1 Hirosawa, Wako, Saitama, 351-0198, Japan

[#]Current address: Institute for Plant Physiology, Justus Liebig University Giessen, Senckenbergstrasse 3, D35390 Giessen, Germany

[*] To whom correspondence should be addressed. Tel: +81 (0)48-467-4598; Fax: +81 (0)48-462-4701; Email: heddle@riken.jp

Supplementary Information

Supplementary methods

In-gel digestion and MALDI-TOF MS

Protein samples were resolved on an 8% SDS-PAGE and the protein bands were visualised by staining with Coomassie blue. The target gel bands were excised, destained and digested with 0.03 mg trypsin (TPCK-treated, Worthington Biochem. Co.) at 37 °C for 12 h in 20 mM Tris·HCl (pH 8.0). The resulting peptides were applied to MALDI-TOF MS on an ultrafleXtreme TOF/TOF MS (Bruker Daltonics, Bremen, Germany) in reflector mode using α-cyano-4-hydroxycinnamic acid as the matrix[1].

Western blots

The dimerised GyrB proteins samples in the native PAGE were transferred onto a PVDF membrane using the semi-dry "trans-blot turbo" system (Bio-Rad). The membrane was then incubated in blocking solution (3% non-fat milk), anti-6x histidine antibody solution (1:2500, Wako) and goat anti-mouse antibody solution (1:5000, Enzyme Research Laboratory) using the SNAP i.d. system (EMD Millipore). The signals were visualised by adding a chemiluminescence substrate (GE), observed using a LAS3000 imager and analyzed by ImageJ[2].

Sequence alignments

Amino acid sequences of proteins were aligned and viewed using CLC Sequence Viewer (www.clcbio.com) with the default parameters.

References

1 Uematsu, S. *et al.* N-Glycosylation of extracellular matrix protein 1 (ECM1) regulates its secretion, which is unrelated to lipoid proteinosis. *FEBS Open Bio* **4**, 879-885, (2014).

2 Schneider, C. A., Rasband, W. S. & Eliceiri, K. W. NIH Image to ImageJ: 25 years of image analysis. *Nat. Methods* **9**, 671-675, (2012).

3 Larkin, M. A. *et al.* Clustal W and Clustal X version 2.0. *Bioinformatics* **23**, 2947-2948, (2007).

Supplementary Figure Legends

Supplementary Figure S1: Expression and purification of TgGyrA and TgGyrB proteins. (a) TgGyrA and TgGyrB were produced and purified separately and samples analyzed using SDS-PAGE. (b) TgGyrA and TgGyrB expressing cells were mixed before affinity purification. The co-eluted TgGyr complex was collected and analysed by SDS-PAGE (lane "TgGyr"). Control lane "EcGyr" shows a mixture of individually purified EcGyrA and EcGyrB. Protein samples (2 μg) were resolved on an 8% SDS-PAGE gel and revealed by Coomassie blue staining. Protein marker sizes (kDa) are indicated. Bands marked with an asterisk were subjected to MALDI-TOF protein identification and confirmed as TgGyrA and TgGyrB.

Supplementary Figure S2: DNA supercoiling activity of a TgGyrB-EcGyrA or TgGyrA-EcGyrB hybrid gyrase. Lane one shows the starting relaxed DNA substrate. EcGyrA and EcGyrB were 33 nM (+) and 66 nM (++) respectively, TgGyrB was 18 nM (+) or 36 nM (++) and TgGyrA was 6 nM (+). OC = Open circular DNA, R = Relaxed DNA, SC = Supercoiled DNA.

Supplementary Figure S3: Western blot of the results shown in Figure 1e using an anti-His tag antibody.

Supplementary Figure S4: Part of the sequence alignment of GyrB from *E. coli*, *M. tuberculosis*, *T. gondii* and *P. falciparum.* Sequences were aligned using the CLC Sequence Viewer. Asterisks denote the residues known to be involved in dimerisation (Tyr-5 and Ile-10).

Supplementary Figure S5: Part of the sequence alignment between EcGyrA, MtGyrA and TgGyrA. The EcGyrA tail residue numbers are 838-875 and the MtGyrA tail residue numbers are 817-838. * is the defined end of C-terminal structure in EcGyrA and MtGyrA. No clear evidence of a GyrA tail is found in TgGyrA.

Supplementary Figure S6: Sequence alignment of GyrA from *E. coli* (1-875), *M. tuberculosis* (1-838) and *T. gondii* (259-1594: lacking signal/transit sequence). Sequences were aligned using the CLC Sequence Viewer (www.clcbio.com).

Supplementary Figure S7: Sequence alignment of regions of full-length mature sequences of TgGyrA, MtGyrA and EcGyrA using ClustalW[3]. (a) A region of the alignment including the QRDR shows that TgGyrA has a quinolone resistance mutation at position 83 (Ser in EcGyrA, marked with an asterisk). (b) A region of the alignment including the Ca^{2+} binding motif in MtGyrA (highlighted by the black boxes). No such sequence is present in TgGyrA. (c) A region of the alignment the MtGyrA-box-l motif in MtGyrA and TgGyrA (highlighted by the black box). No such sequence is present in EcGyrA.

Supplementary Figure S1

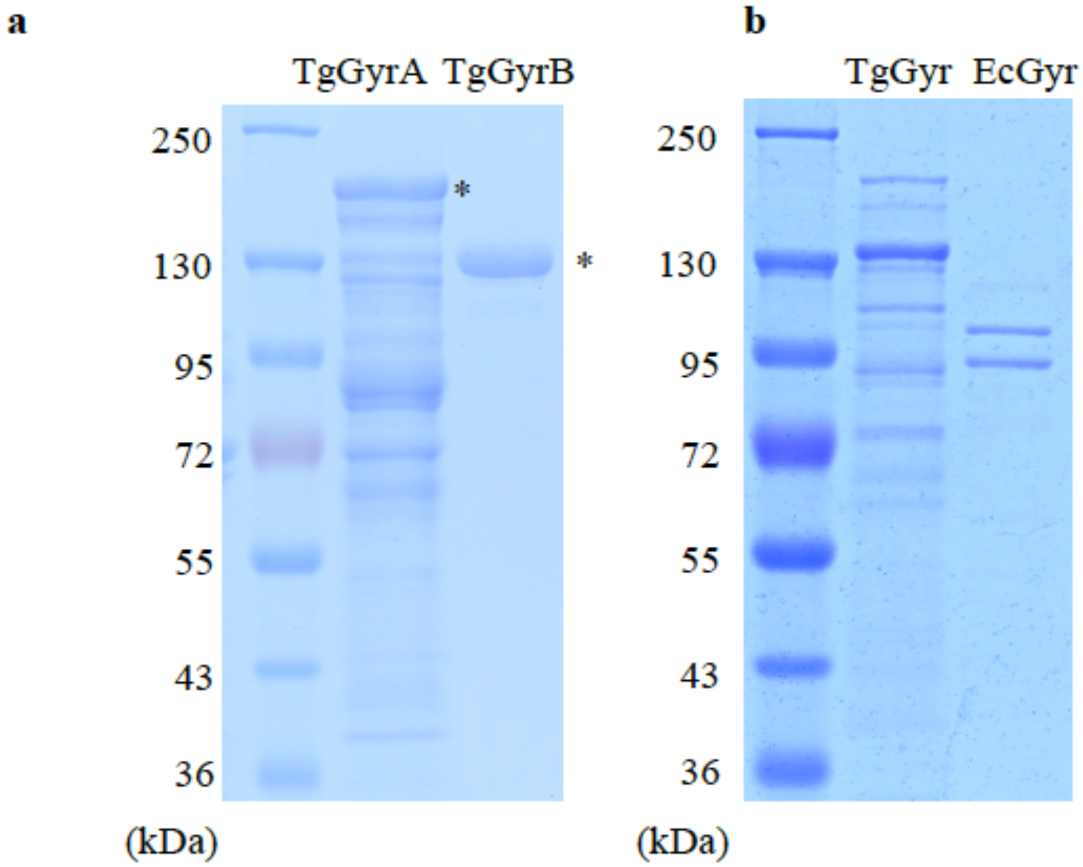

Supplementary Figure S2

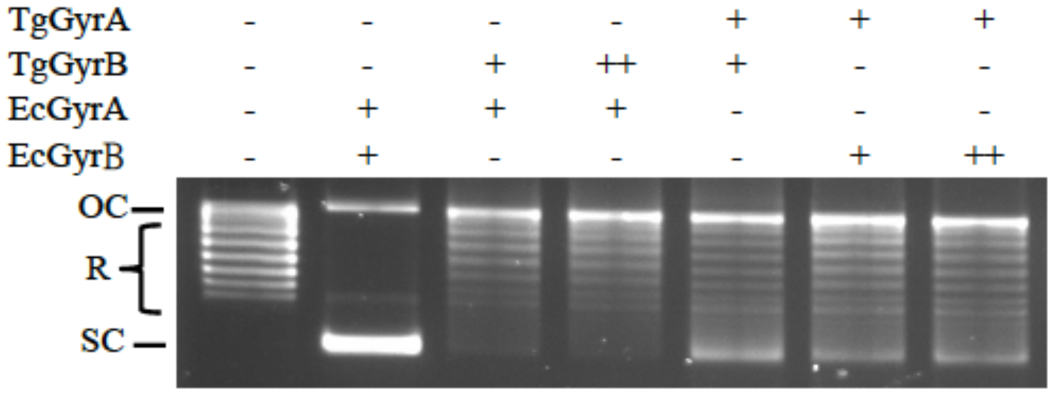

Supplementary Figure S3

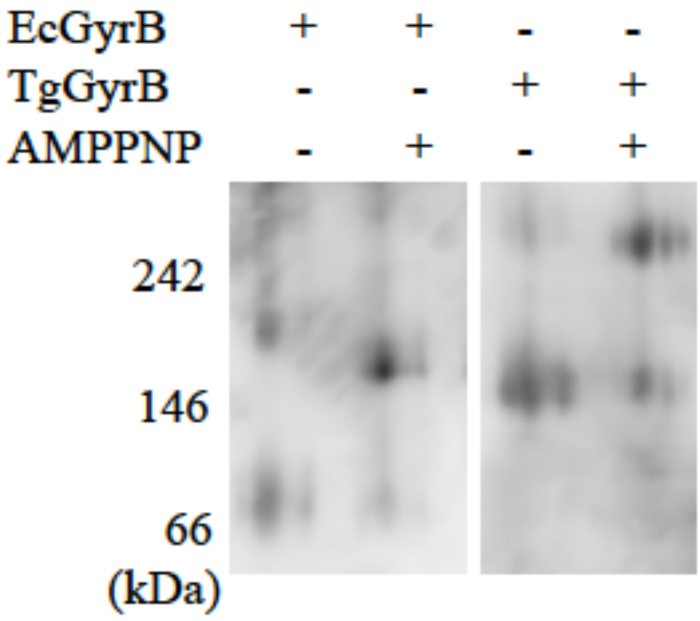

Supplementary Figure S4

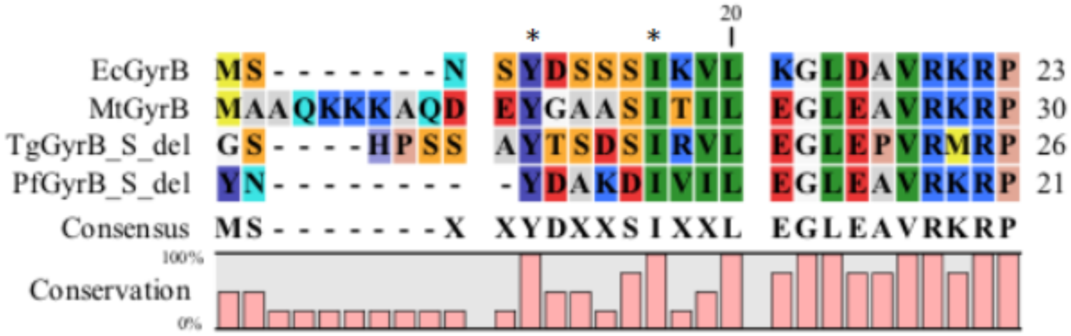

Supplementary Figure S5

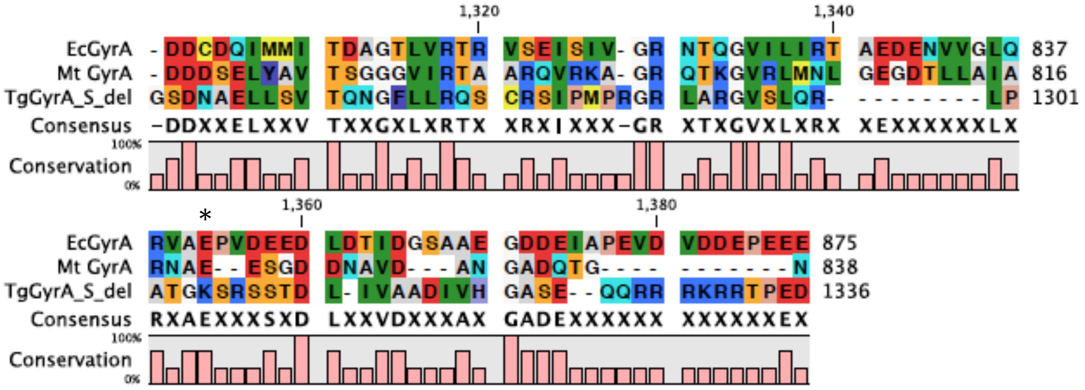

Supplementary Figure S6

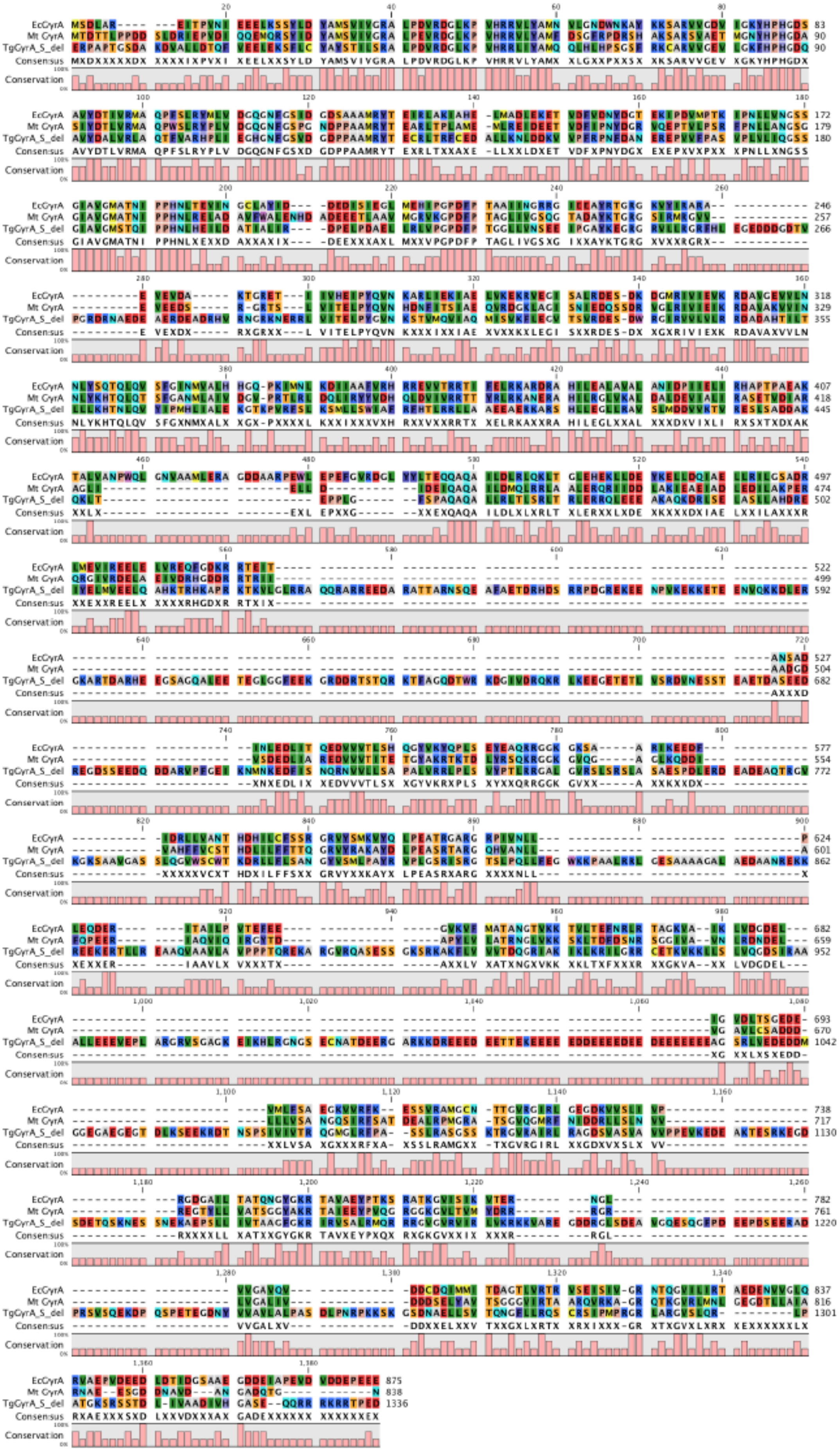

Supplementary Figure S7

a

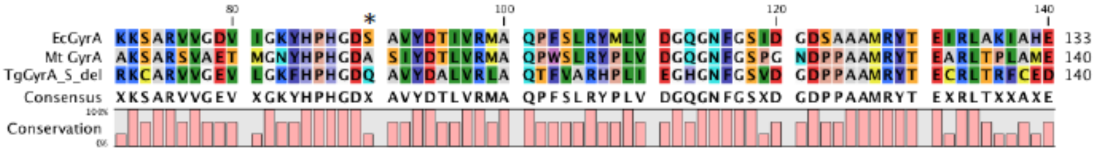

b

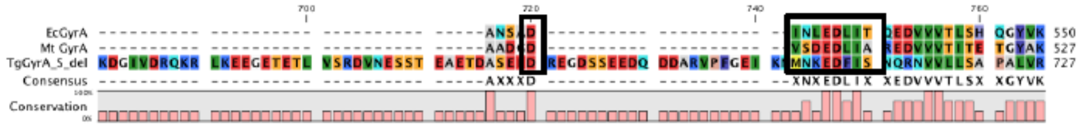

c

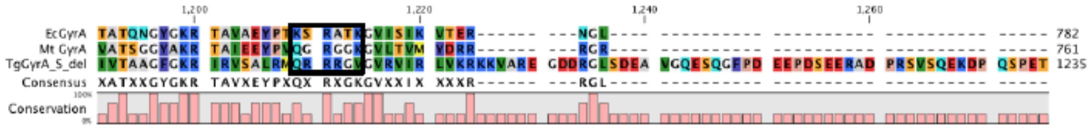

Supplement: Supplementary Information [file srep14491-s1.pdf]
